# Scaffold-based bone tissue engineering in microgravity: potential, concerns and implications

**DOI:** 10.1038/s41526-022-00236-1

**Published:** 2022-10-29

**Authors:** Federico Mochi, Elisa Scatena, Daniel Rodriguez, Maria-Pau Ginebra, Costantino Del Gaudio

**Affiliations:** 1 E. Amaldi Foundation, Via del Politecnico snc, 00133 Rome, Italy; 2 Biomaterials, Biomechanics and Tissue Engineering Group, Department of Materials Science and Engineering, Universitat Politècnica de Catalunya (UPC), Av. Eduard Maristany 10, 08019 Barcelona, Spain; 3 Barcelona Research Center in Multiscale Science and Engineering, Universitat Politècnica de Catalunya, Av. Eduard Maristany 10, 08019 Barcelona, Spain; 4 Institute for Bioengineering of Catalonia (IBEC), Barcelona Institute of Science and Technology (BIST), Baldiri Reixac 10, 08028 Barcelona, Spain

**Keywords:** Fracture repair, Cell biology

## Abstract

One of humanity’s greatest challenges is space exploration, which requires an in-depth analysis of the data continuously collected as a necessary input to fill technological gaps and move forward in several research sectors. Focusing on space crew healthcare, a critical issue to be addressed is tissue regeneration in extreme conditions. In general, it represents one of the hottest and most compelling goals of the scientific community and the development of suitable therapeutic strategies for the space environment is an urgent need for the safe planning of future long-term manned space missions. Osteopenia is a commonly diagnosed disease in astronauts due to the physiological adaptation to altered gravity conditions. In order to find specific solutions to bone damage in a reduced gravity environment, bone tissue engineering is gaining a growing interest. With the aim to critically investigate this topic, the here presented review reports and discusses bone tissue engineering scenarios in microgravity, from scaffolding to bioreactors. The literature analysis allowed to underline several key points, such as the need for (i) biomimetic composite scaffolds to better mimic the natural microarchitecture of bone tissue, (ii) uniform simulated microgravity levels for standardized experimental protocols to expose biological materials to the same testing conditions, and (iii) improved access to real microgravity for scientific research projects, supported by the so-called democratization of space.

## Introduction

It is well-stated that reduced gravity, as experienced during space missions, significantly affects human physiology leading to, among others, muscle atrophy, reduction in bone density and immune function, endocrine, and blood disorders. Induced modifications also contribute to possible tissue damage that could occur in such a harsh environment^1,2^, and the extent of the affections is directly related to the temporal exposure to microgravity. These factors, nowadays, represent the major obstacles to a safe long-term space mission which imply the need for the crew to be prepared as an autonomous and self-consistent unit capable of dealing with all the possible drawbacks related to healthcare management. In this regard, a particular attention should be paid to tissue engineering and regenerative medicine with the aim to exploit the potential of this interdisciplinary approach in different gravity conditions, i.e., Moon, Mars, and the International Space Station (ISS), being the next and the current scenarios of space exploration. In order to focus on this specific topic, the present review discusses the data collected so far in the field of scaffold-based bone tissue engineering in microgravity and explores the perspectival applications for space missions. Several studies have been performed on cell cultures in microgravity, simulated or not, but most of them refer to isolated cells exposed to this environmental input evaluating the adaptive response. In this regard, the proposed assays can be affected by simplified in vitro models as the role of the extracellular structures is generally not taken into account, being then minimally compliant to the physiological characteristics that, in turn, contribute to define a specific biological output. The influence of a biomimetic means capable to replicate the properties of the natural cell microenvironment, ranging from morphological to biochemical cues, can offer the possibility to collect more detailed results and, for this aim, scaffold-based microgravitational bone investigations are here considered.

**Fig. 1:**
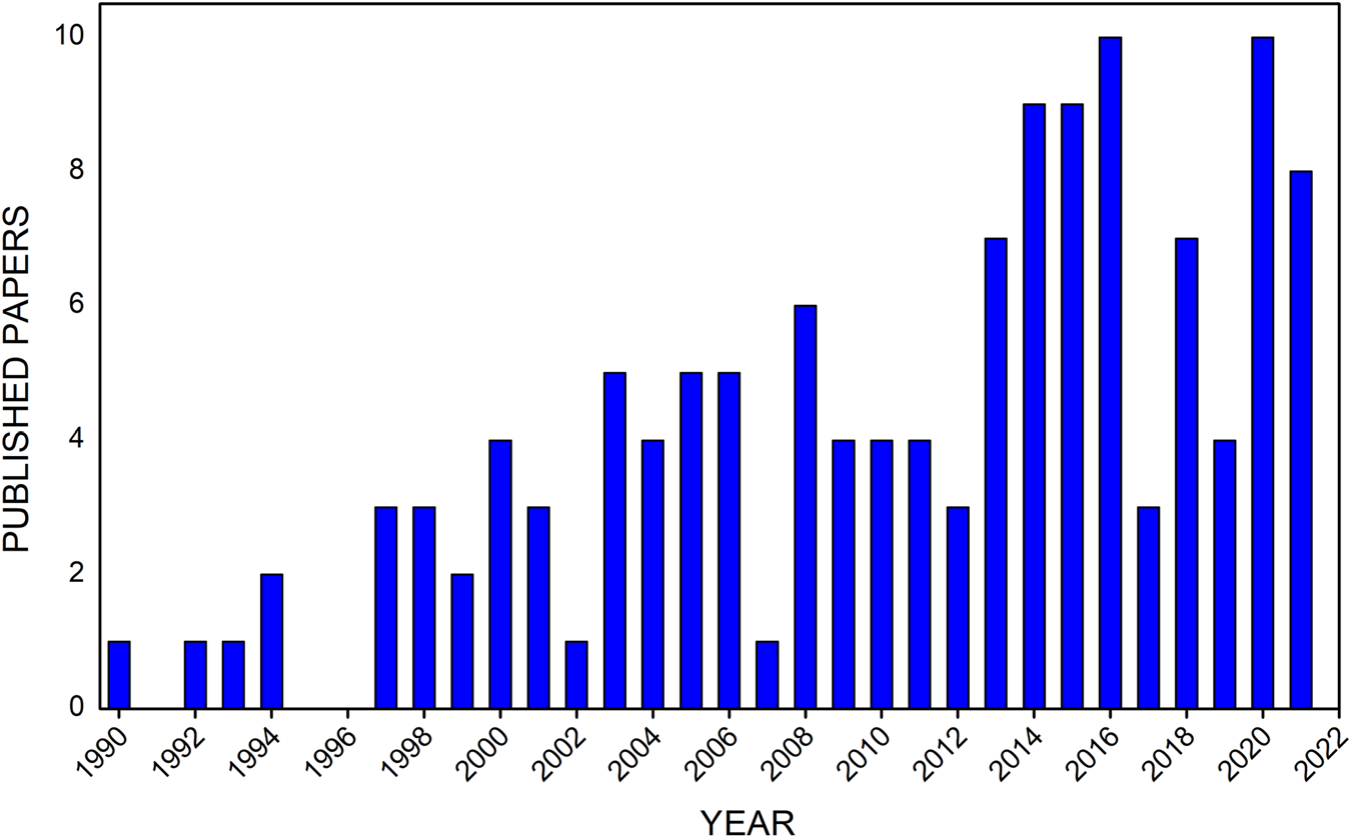
Papers dealing with bone tissue engineering in microgravity. Timeline of published papers from the PubMed website using the key “microgravity bone tissue engineering”.

The preservation of the skeletal system is one of the main concerns for human spaceflight as microgravity modifies the normal bone remodeling rates, causing resorption (osteoclast activity) at a faster rate than ossification (osteoblast activity). Bone loss is elicited within days of unloading and an in-depth evaluation of the functional response of bone tissue is therefore mandatory in order to address and deal with a number of issues, including, e.g., the role of spaceflights on bone healing, the factors that influence bone strength, and the necessary countermeasures to be developed^3^. The relevance of this investigation can be promptly highlighted in Fig. 1, showing the increase in published papers referring to microgravity-related bone concerns. Since 1990, however, no more than 130 reports have been collected in the PubMed database. Such a limited result can be related to the intrinsic complexity of the space environment and to the obvious restrictions for direct access that contribute to define a circumscribed research area. However, bone tissue engineering in space should be considered a field of practical interest in view of the programmed long-term/long-distance missions.

Space missions are characterized by extremely high costs and research in the near-Earth orbit is severely constrained by the limited number of flight opportunities. For this reason, different systems to simulate microgravity (SMG) conditions have been developed. These devices include diamagnetic simulators, 2D and 3D clinostats, rotating wall vessels and random positioning machines^4–6^. The rationale behind the selection of SMG devices should be clearly stated, as each of them induces artifacts in the experiment. It is, therefore, often difficult to conclude whether the biological responses are caused by the simulated microgravity or by any other possible effect due to the simulation technique^7–10^.

With the aim to critically discuss findings on bone tissue engineering in microgravity conditions, especially focusing on the role of the scaffolds to provide a more similar environment to cells and collect a more realistic response, this review is structured as follows. A brief recapitulation of bone pathophysiology related to spaceflight is reported, underlining the bone loss implications; the following section is then dedicated to bone scaffolds with special attention to the materials and their properties for a specific regenerative application, to the design requirements to support a suitable outcome, and to the bioreactors capable to allow 3D cell cultures in simulated microgravity; finally, an analysis on the experimental results acquired both in real and simulated space environments is presented. The rationale of this study is conceived to provide an overview of a very specific research area, highlighting state-of-the-art and, hopefully, support an increasing involvement in the research of gravity-induced physiological modifications in order to develop countermeasures with a potential downstream benefit for ground treatments.

Reasonably, even this proposal can be regarded as a simplified approach to a more complex problem, but the influence of a three-dimensional cell culture scaffold can offer more direct indications of the modified cell response to microgravity-related dysregulations compared to suspended cultures or cell-seeded assays onto two-dimensional substrates.

### Bone physiology and spaceflight-related bone loss

Strength, shape and stability of the human body are dependent on the musculoskeletal system. Bone is a modified form of connective tissue which is made of extracellular matrix, cells, and fibers. The cell lines involved in bone growth are mesenchymal stem (or stromal) cells (MSC), osteoblasts, and osteocytes. MSCs represent a source of new osteoblasts, which line the bone surface, secrete collagen and the organic bone matrix (osteoid), and calcify soon after deposition. Being trapped in the organic matrix, osteoblasts become osteocytes which have the function of maintaining bone tissue. Fine processes from these cells ramify through bone, and form gap junctions with other osteocytes in the calcified matrix, in small spaces called lacunae. Long processes from the osteocyte lie in small channels (canaliculi), providing transport of nutrients and waste^11^.

Bone remodeling is driven by osteoclasts, large multinucleated cells with a ruffled border resorbing bone matrix by secreting enzymes, such as carbonic anhydrase, which acidifies the matrix and causes decalcification and hydrolyzation, thus leading to the final breakdown of the matrix. Osteoclasts, thought to be monocytes derivative, are bigger than osteoblasts and osteocytes and have many vacuoles containing acid-phosphatase enzymes that facilitate bone resorption^12,13^.

Bone remodeling is necessary for growth and is mainly stimulated by the release of calcium and hormones. In physiological conditions, many factors regulate bone formation and resorption, like parathyroid hormone (receptor localized in the osteoclast), calcitonin (receptor localized in the osteoblast), estrogens, vitamin D, various cytokines, and other local factors such as prostaglandins that are all closely balanced. The bone extracellular matrix is also involved in various processes like differentiation, proliferation, and responses to growth factors, and can induce the production and absorption of bone tissue^10^. The bone matrix is a dynamic environment composed of about 65 wt% minerals, 25 wt% organic phases, and the remaining 10 wt% by water^14^. The main inorganic mineral component is hydroxyapatite (Ca_10_(PO_4_)_6_(OH)_2_), while the most abundant organic fraction is a type I collagen (nearly 90%)^15^.

From a structural point of view, two basic types of osseous tissue can be observed in varying proportions, the compact (or cortical) and the spongy (or cancellous or trabecular) bone, made up of trabeculae in the form of small beams, struts or rods with micrometric dimensions^16^. The trabecular bone is localized in the epiphysis of long bones and in the vertebrae body; it is more prone to frequent fractures (due to trauma) and regenerates more quickly than cortical bone. In this regard, trabecular bone is more likely to be affected when the breakdown and regeneration of bone are out of balance in osteoporosis^17^.

Bone loss is a condition observed in bedridden older people, while osteoporosis is a disease that weakens bones, leads to decreased bone density and is diagnosed when a person has a bone mass 30 percent lower than the average young adult. This pathology affects the strength of the bone and makes fractures more likely. Risk factors for osteoporosis include aging, gender (females being more exposed), low body weight, low sex hormones or menopause, smoking, and some medications^18^. Statistical data regard osteoporosis as one of the most widespread diseases and the most frequent cause of fragility fractures for people over 50 years old^19^. Osteoporotic bone loss affects cortical and trabecular bone, decreasing cortical thickness and the number and size of trabeculae (trabeculae may be disrupted or entirely absent) with the consequence to increase porosity and decreased mechanical strength^17^.

Pathological conditions of bone tissue are also elicited by external conditions as a response to environmental input. Bone reacts to mechanical stimuli, and the influence of mechanical forces on the structural development and remodeling of bone has been long established^20^. External mechanical forces are a potent regulator of MSCs differentiation in vitro^21^. In this respect, the accelerated loss of bone and muscle mass as a result of microgravity exposure has been well documented over decades^22,23^. It has been demonstrated that microgravity induces several detrimental effects on the equilibrium of osteoblasts and osteoclasts, leading to a more intense activity of osteoclasts^24^. Peripheral quantitative computed tomography analysis of distal tibia in astronauts, before and after spaceflights, shows up to 24% of trabecular bone lost after six months from the end of the mission^25^ and the retrieval of normal skeletal density after a long duration space mission was estimated to be about 3 years^26^. The low gravity effects experienced during long-term spaceflights, therefore, disrupt skeleton homeostasis, causing a release of calcium in blood at a rate that is almost ten times greater than that in a postmenopausal woman^27^. This physiological adaptation implies a higher risk of fractures and potential long-term health risks for astronauts on their return to Earth^28^. Countermeasures mainly consist of exercise and supplementation of calcium and vitamin D, which need to be assessed in detail. For instance, an additional benefit of performing exercise in space has profound effects on the normal functioning of the immune system, but intensity and duration need to be finely balanced as frequent heavy, prolonged bouts of endurance exercise can be related to the immune system impairment, latent viral reactivation and increased infectious episodes; regarding vitamin D, it is well-known that regulates calcium homeostasis on bone formation and resorption, but also affects other biological processes including modulation of the immune system as well^29^. Recent researches about the genes involved in osteoporosis and in bone homeostasis status provide the basis for the development of therapeutical strategies for astronauts^30^.

**Fig. 2:**
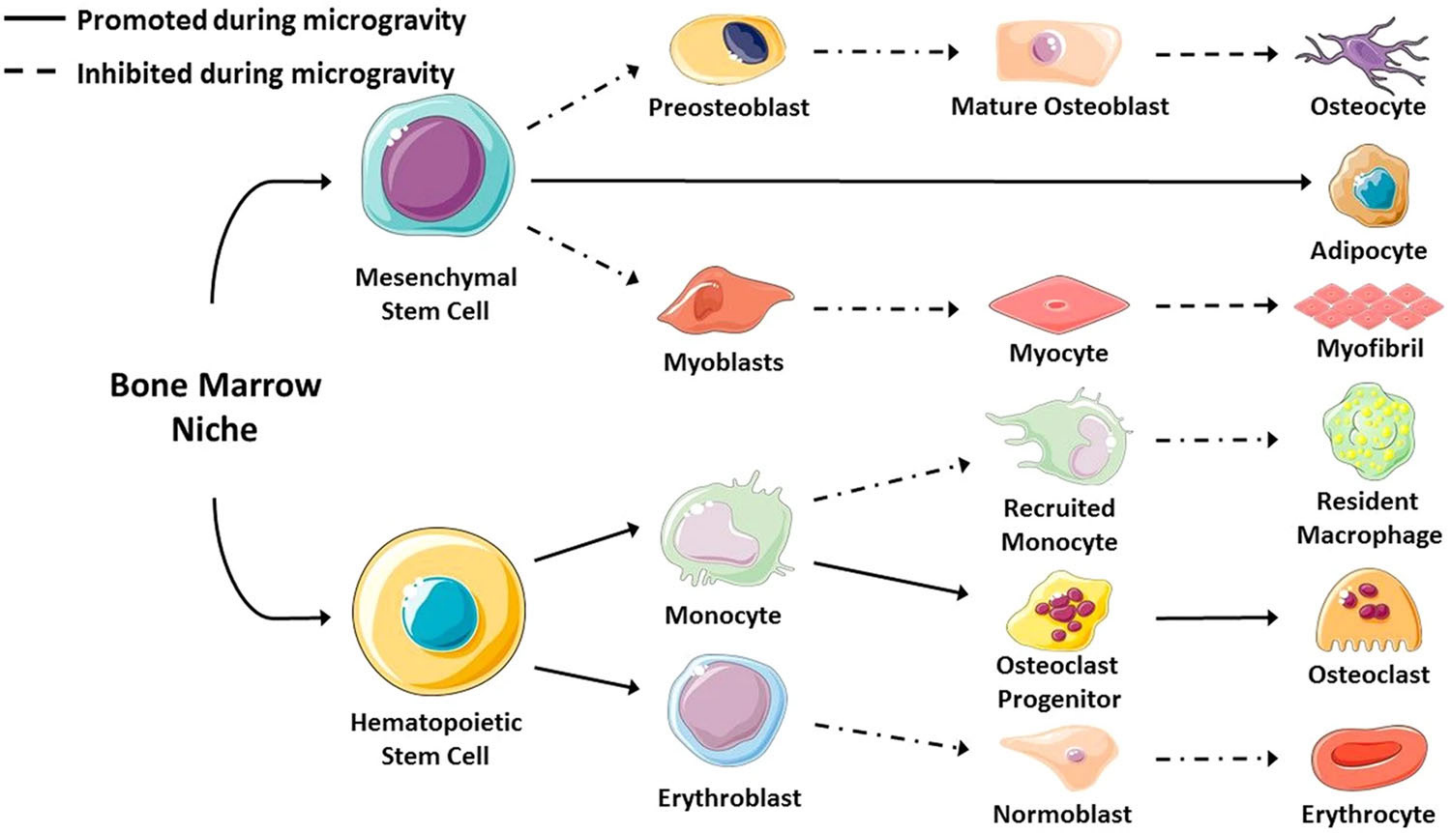
Differentiation of cell lines depending on gravity. The faith of the cells is strongly affected by gravity. Mesenchymal stem cells mainly differentiate in adipocytes, while hematopoietic stem cells move toward the osteoclast pathway^3^ (in compliance with CC BY 4.0 License— https://creativecommons.org/licenses/by/4.0/).

In this scenario, aiming at possibly linking the research results from space to Earth, and vice versa, it is possible to draw some considerations on the differences and analogies of osteopenia experienced in these two extremely different environments. Osteopenia is a skeletal condition characterized by weaker bones due to an impaired bone regenerative mechanism. Normally, osteopenia can be related to genetic factors, overacting thyroid or untreated celiac disease and is more probable in the female sex, especially in the postmenopausal age. Specific medical treatments can also concur to its onset, like long-term steroid administration, antiseizure drugs, and anticancer radiation therapy. Some lifestyle factors could also improve the risk of developing osteopenia, such as a lack of vitamin D, smoking, or a bedrest condition^31^. During a spaceflight, the absence of gravity may predispose astronauts to the early risk of osteoporosis, because atypical changes (for healthy persons) are observed, such as the increased excretion of calcium, the reduction of bone mineral density, especially for the trabecular part, the accelerated rates of bone loss, and the delayed recovery of hip trabecular bone^32–34^. Although osteopenia occurs for different causes on Earth and during spaceflights, from a histological point of view, the reduction of trabecular bones have some qualitative similarities compared to menopause-induced bone loss, as common characteristics of lack of estrogen and mechanical loading include the reduction of bone density, an increased rate of bone resorption, a decrease in bone strength, and a perturbed endocrine regulation. The vertebrae, hip, and wrist are more vulnerable to developing osteoporosis due to the high content of cancellous bone and cortical thinning, and the same skeletal districts are most susceptible to fracture during activities performed on space missions^35^. In addition, focussing on the cell response, it can be remarkably underlined that exposure to microgravity leads to bone marrow stromal cells to preferentially differentiate to adipogenic lineage cells instead of the osteogenic pathway (Fig. 2). This outcome impairs their potential to respond and adapt to the mechanical environment, and to regenerate and recover bone tissue from injuries, also decreasing cell population and function. This is similar to what is seen with aging, and it has been therefore suggested that investigating the effects of microgravity exposure can contribute to the comprehension of aging effects^3^.

On Earth, several pathological affections can benefit from novel clinical treatments supported by, e.g., tissue engineering and regenerative medicine (TERM) procedures. This model can be replicated in space, deeply investigating all the key elements that concur with the desired output, ranging from the materials to the fabrication techniques. To confirm this statement, for instance, 3D bioprinting is considered by the European Space Agency (ESA) a long-term enabling technology for distant planet exploration and colonization^36^. Therefore, the potential of TERM methodologies deserves a special focus in order to allow crew members to deal with serious medical contingencies as a safe return to Earth could not be timely ensured, depending on mission duration, medical personnel and support infrastructure availability^36^.

## Scaffolds for bone tissue engineering

Tissue engineering combines several disciplines, such as materials science, engineering, and clinical medicine^37^. Its goal is to restore the function of damaged tissues, representing a therapeutic alternative to autografts. Properly designed scaffolds are substitute materials mimicking the physical and biochemical properties of the healthy tissue, and providing a 3D environment to promote cell adhesion, proliferation and differentiation to restabilize the physiological function of a tissue. Scaffolds, cells, and growth factors are known as “the tissue engineering triad”^38^, and some authors consider the extracellular matrix the fourth factor^10^. An ideal scaffold for bone tissue engineering should be biodegradable, biocompatible, bioactive, osteoconductive, and osteoinductive^39^. However, all these considerations should be further stressed referring to space due to a possible modification of the tissue engineering approach toward a gravity-dependent model in which the degenerative effects should be fully elucidated and a mitigation strategy planned, as a consequence^40^.

### Materials

A large number of biomaterials is currently used for the fabrication of bone tissue scaffolds, including natural or synthetic polymers and inorganic materials. Each material presents advantages or disadvantages, due to their nature, and the selection of a biomaterial is driven by the closeness of the biomechanical properties to those of the implantation site^41^.

Naturally-derived biomaterials (e.g., collagen, gelatin, chitosan, starch) can offer several active cues to cells, even if may elicit immunogenic issues and the biodegradability rate can be difficult to control^42^. Synthetic polymers, e.g., polylactic acid (PLA), polycaprolactone (PCL), or polyurethane (PU), are more frequently considered due to the higher control on the degradation rate and mechanical properties^43–45^. Metals (e.g., titanium alloys) or inert ceramics (e.g., alumina and zirconia) can be suitable options characterized by high strength and biocompatibility, but with a limited interest in tissue engineering applications due to their non-degradability. To overcome this limitation, the use of biodegradable metallic alloys, such as magnesium-based, iron-based, and zinc-based alloys, has been proposed^46^. Mg-alloys are the most promising bioresorbable metallic biomaterials for tissue engineering applications. Magnesium is present in large amounts in the human body and it is essential for many physiological processes, especially in bone tissues. About 67% of Mg is stored in bone tissues, while 30% is exchangeable on the crystal surface of the bone and contributes to maintaining intra- and extracellular Mg concentrations. Magnesium ions (Mg^2+^) influence the overall rate of seeded calcium phosphate crystallization and the subsequent growth of hydroxyapatite, contributing to induce osteogenic and osteoblast differentiation, thereby promoting bone regeneration^47^. For bone tissue engineering purposes, Mg and its alloys can be considered both as a filler^48^ or as the structural material of scaffolds^49–51^.

Bioactive ceramics (e.g., hydroxyapatite (HA), tricalcium phosphate, bioactive glasses) and their combinations, because of their similarities with the mineral phase of bone, have been widely used, even though it must be borne in mind that biological HA is non-stoichiometric and, besides being calcium deficient, it contains several impurities, mainly carbonates but also other anions and cations^52,53^. The introduction of calcium phosphate cements (CPCs) in the early 1980s provided clinicians with moldable and even injectable pastes that were able to harden within the body. Formulations were developed and apatite cements were proposed based on other compounds like alpha TCP (α-TCP), and also cements that produce other final phases like brushite (DCP dihydrate, DCPD) or monetite (DCP anhydrous, DCPA). The wide range of formulations allows users to adapt its properties to specific clinical needs and requirements for different degrees of resorbability, and, most interestingly, allows to deal with these pastes for the fabrication of biomimetic scaffolds by means of foaming or 3D-printing techniques such as robocasting. The hardening of the cement is a non-exothermic reaction, with the formation of a porous network of micrometric/nanometric CaP crystals^52^.

CPCs are a model bioceramic for doping and ion-doped CPC is often superior to the non-doped option in its osteogenic functions^54^. The most biologically relevant anionic substitutions include carbonate for the phosphate or OH^−^ groups, and F^−^ and Cl^−^ for the OH^−^ group^55^. Magnesium-substituted HA (MgHA) has also drawn interest due to the vital role of Mg in stimulating osteoblast proliferation during the early stages of osteogenesis^56^.

From a functional point of view, scaffolds produced from a single biomaterial may present a number of drawbacks due to the peculiar characteristics of the biomaterial itself (as summarized in Table 1). Composite biomaterials are designed to combine two or more different materials with the goal to improve the processability, mechanical properties and bioactivity of the final scaffolds with the aim to further resemble specific characteristics of the target tissue as biological tissues can be considered natural composites^57^. In the case of bone, a collagen matrix is reinforced with bioceramic (Ca-deficient HA) nanoparticles, and can be thus treated as a ceramic-organic bionanocomposite system^58^. The mechanical properties of bone are dependent on its composition and structure, and an understanding of the structural behavior is extremely important for the evaluation of fracture risk. Composite bone scaffolds may combine inorganic materials like bioceramics with natural polymers, such as collagen, in order to mimic the composition and mechanical properties of bone.

**Table 1:** Most used materials for bone tissue engineering.

	Materials	Advantages	Disadvantages
Natural polymers	Collagen	Osteocompatible, easily embedded into the tissue matrix	Low mechanical strength, adverse immunological response
	Gelatin	Versatile and relatively low cost, porous structure of gelatin scaffolds can be easily tailored	Poor mechanical properties
	Chitosan	Biodegradation rate can be tailored by the amount of residual acetyl content, chemical modifications of chitosan adjust physical properties, antimicrobial activity	Poor mechanical properties
	Hyaluronic acid	Easy chemical modification, non-antigenic, injectable	Rapid degradation
	Agarose	Osteoconductive, hemostatic, injectable	Poor mechanical properties
	Alginate	Wide range of gelling, biocompatibility, easy chemical modifications	Purification needed to remove possible mitogenic, cytotoxic and apoptosis-inducing impurities
Synthetic polymers	Polylactic acid (PLA)	Good processability, cost-effective	Hydrophobic, slow degradation
	Poly-Ɛ-caprolactone (PCL)	Very good processability, inexpensive, easily blended with other polymers	Slow degradation
	Polyurethane (PU)	Excellent mechanical properties	Precursors have to be accurately chosen to obtain PU with low-toxicity degradation products
Bioceramics	Bioactive glasses	Bioactive, improve cell differentiation	Brittle
	Hydroxyapatite (HA)	Bioactive, non-inflammatory	Slow degradation, brittle
	Tricalcium phosphate (TCP)	Osteoconductive, support of osteogenic differentiation in vivo	Slow degradation, Brittle
Metals	Magnesium alloys	Osteogenesis enhancement, immunomodulation, angiogenesis	Corrosion by-products (H_2_ production, alkalinization)

Composites can be a valid option to create scaffolds tailored for an indented use and compliant to a biomimetic design, i.e., being capable of closely replicating the properties of living tissue. Successful biomimetic biomaterials can ideally restore the natural function of injured tissue or organ if the concurrent scaffold degradation does not release toxic by-products. A number of combinations of biomaterials for bone scaffolding with nanoscale features have been tested following this philosophy (Table 2). Many commonly used composites have mechanical properties similar to trabecular bone, even though they have not allowed to replicate the multiscale structure of bone^59^. A significant issue for the production of these composites is the processing methods used in their manufacture and their relative ease of application in space.

**Table 2:** Composites for bone scaffolding.

Inorganic material	Organic material	Processing	Results	References
HA	Silk fibroin	Biomineralization	Enhanced physical and chemical properties such as cell activity and osteogenesis	^111,112^
HA	PLA nanofibers	Electrospinning	Improved osteogenesis in HA-PLA fibers with respect to neat PLA fibers	^113,114^
HA	Gelatin, polylactide-co-caprolactone (PLCL)	Precipitation	Composites promoted mineral, cell attachment and proliferation	^115^
HA	Collagen + chitosanhydrogel, collagen + alginatehydrogel	Electrodeposition	Improved bioactivity as tested on MC3T3-E1 cells	^116^
HA	Polycaprolactone (PCL)	Electrospinning + electrospraying	Accelerated in vivo bone regeneration	^117,118^
HA	Polycaprolactone (PCL) + biomimetic calcium phosphate–coated poly(caprolactone) nanofiber (BCP)	Electrospinning + biomimetic mineralization	BCP constructs provided a more favorable environment for ECM production	^109^
HA	Collagen, glycosaminoglycan	Self-assembly	Good mechanical and thermal properties, scaffolds promoted osteogenic differentiation and proliferation	^119^
HA	Crosslinked Poly-etylenglycol (PEG)-poly(urethane-urea)	Crosslinking + physical blending	Higher cell adhesion and density; improved biomineralization	^120^
HA	Poly-hydroxy-butyrate (PHB)	Electrospinning	Osteoid tissue formed in vivo. Scaffold absorbed in 2 months after the implantation	^121^
Sr(2+)/Fe(3+)co-doped HA	Decellularized intestinal mucosa	Self-assembly + crosslinking	Cellular functionality and bioactivity of endotheliocytes/osteoblasts were significantly enhanced	^122^
HA	Polyamide-6/chitosan	Electrospinning	Non-cytotoxic, good biocompatibility, supported cell attachment, and proliferation.	^123^
HA+ graphene oxide	Collagen	Molding + biomimetic mineralization	The mineralized composites exhibited loose porous structures with excellent antibacterial properties and biocompatibility	^124^
HA	Collagen/epigallocatechin gallate	Freeze-drying + coating	Composite sponges promoted cell adhesion and proliferation	^125^
HA/bioglass	Collagen	Ultrasound-assisted sol-gel method + biomimetic mineralization	Improved physicochemical and mechanical properties; enhanced in vivo bone formation	^126^
HA	Collagen	Crosslinked hydrogel + freeze-drying	Composites promoted osteogenic differentiation of MC3T3-E1 cells	^40,127^
Bioglass	Polypropylene fumarate, hydroxyethyl methacrylate (PPF/HEMA)	Crosslinking + biomineralization + biodegradation	Good adhesion and viability of osteoblast cells	^128^
Bioactive glass nanoparticles	Polycaprolactone (PCL)	Freeze-casting + oxygen plasma + freeze-drying	Interconnected lamellar channels provided nutrient supply and ion exchange, enhancing cell proliferation	^129^

### Manufacturing technologies

Several techniques have been implemented so far to produce 3D scaffolds for bone tissue engineering. One of the goals of these methodologies is to provide an interconnected 3D pore structure that allows cell attachment, proliferation, and differentiation (if stem cells) as well as the transport of waste products and metabolites^60^. A number of studies have been performed on the effect of average pore size on cell diffusion within the scaffolds and bone growth, establishing that a minimum open porosity over 100 µm is required^61^. Additionally, the degradation of the scaffold should ideally match the regeneration of the healing tissue^62^.

#### Solvent casting and particulate leaching

This technique is used to produce highly porous scaffolds. The first step is the dissolution of the polymer in a solvent, mixing the solution with a soluble porogen, and then casting it in a Petri dish. After solvent evaporation, the composite is leached in a selective solvent to remove the porogen. The resulting scaffold porosity can be controlled by the amount of porogen added, while the pore dimension depends on the size of the crystals^63^. Different porogens have been tested as waxy hydrocarbons, sodium chloride, or gelatin particles^64^. The major problem of this technique is the possible residual amounts of organic solvent in the resulting scaffold that can cause a decrease in the activity of bioinductive molecules and cell response^65^. However, also pore size can represent a further limitation to be assessed in order to deal with a suitable scaffold providing cell adhesion, proliferation and migration. A design trade-off should be critically considered between efficient mass transport and optimal structural functionality during the remodeling period, which is pivotal for load-bearing structures like bones.

#### Foaming process

A porous scaffold can be prepared by mechanical foaming, gas foaming or a combination of techniques. Mechanical foaming is achieved by whipping (with a mixer, ultrasonic waves or an equivalent method) a mixture of the biomaterial with a foaming agent (typically emulsifiers) that maintains the foamed structure for a reasonable period of time. Adjustment of the mechanical energy used and the concentration of the foaming agent allows controlling the final porosity of the scaffold^66,67^.

In the gas-foaming technique, pores are created by gas expansion without using solvents. This method leads to highly porous foams with a pore size of ~100 µm. As a porogen, gas carbon dioxide is the most used due to its low toxicity^68^.

#### Thermally-induced phase separation

A polymer is dissolved at a high temperature and the mixture is then cooled, obtaining a porous scaffold. The porous structure is generated by phase separation of the components with a resulting pore size between 10 and 100 µm^69^.

#### Solid free-form fabrication technique

Fabrication techniques like stereolithography (SL), fused deposition modeling (FDM), selective laser sintering (SLS), and robocasting are included in the additive manufacturing sector, based on a computer-aid design (CAD) model to produce the 3D scaffold^70,71^. Reasonably, each 3D-printing methodologies is characterized by several pros and cons. Regarding the advantages, for instance, the use of CAD allows to develop highly reproducible structures in terms of shape and porosity by means of automatically controlled processes. 3D-printing technologies have enabled the production of scaffolds with greater spatial resolution than traditional fabrication methods, providing complex porous hierarchical structures^72–75^. Moreover, the development of biomimetic self-setting ceramic inks has circumvented the problems associated with shrinkage in ceramic parts and has allowed nanostructured calcium phosphate scaffolds to be obtained^76^.

Among the possible disadvantages, specifically referred to the techniques, SL can be affected by secondary non-desired photopolymerization, and the materials and the equipment are generally expensive^77^; FDM can process a limited range of suitable materials, being the fabrication temperatures in the range of 200–270 °C, and SLS is based on the laser-induced powder sintering, requiring complex and bulky machinery and producing components with small pores (1–10 µm)^78^.

#### Microsphere sintering

3D porous scaffolds (about 40% porosity) can be fabricated starting from microspheres of different materials, such as ceramics and polymers, realized by solvent evaporation^65^. Microspheres can be assembled into microsphere-based scaffolds following one of the three main packing strategies: random packing (i.e., in a nonspecific manner), directed assembly (by the establishment of cohesive forces), and rapid prototyping (layer-by-layer assembly of microsphere-based scaffold via CAD to create specific architectures)^79^.

#### Emulsion freeze-drying method

Freeze-drying allows to obtain scaffolds with ~90% porosity and pore sizes from 20 to 200 µm^80^. Operatively, a water phase is added to a polymer solution to form an emulsion. Then the emulsion is transferred into a freezer to be solidified for the subsequent freeze-drying process to remove water and obtain the dried scaffold^81^.

#### Electrospinning techniques

The electrospinning technique allows to collect fine fibers with a simple experimental set-up: a spinneret, a syringe pump, a high-voltage power supply, and a grounded collector. It is an electrohydrodynamic process, during which a liquid droplet is electrified to form a jet, experiencing stretching and elongation to generate fibers made of different materials. Fiber diameter depends on the spinning parameters and solution concentration; an increase in solution concentration results in fibers with larger diameters. Typically, the surface of electrospun fibers is smooth and the voids (pores) in the textile structure are large and fully interconnected. To enhance porosity, fibers can be fabricated by electrospinning a suspension of porogens (i.e., salt or clay particles) in the polymer solution, followed by selective porogen removal^82^.

#### Three-dimensional bioprinting

Similar to other solid free-form fabrication techniques, bioprinting starts from a CAD model to produce 3D scaffolds processing a bioink which provides the biological components, such as living cells or biomaterials^83^.

The most common materials for 3D bioprinted scaffolds are hydrogels, but only a few of them are printable^84^. Indeed, a number of requirements have to be considered for suitable bioinks, such as viscosity, shear thinning, surface tension, swelling and gelation kinetics. In addition, bioinks should allow a transition from a liquid state to a gel structure without damaging cell viability^85^. To preserve the biomechanical stability of the scaffold in the post-processing steps, the bioink is often crosslinked (physical, chemical, or enzymatic).

Several techniques of bone bioprinting are available: inkjet, extrusion, and light-based^86^. A critical point to deal with an effective bioprinted tissue is the design and incorporation of a vascular network in the production process. Vascularization is pivotal to guarantee oxygen and nutrients and avoid tissue necrosis^87^, as 3D bone substitutes with poor vascularization could fail after implantation^85^.

As a general comment referring to potential and safe implementation in space, however, each fabrication technique should be carefully evaluated. Porogen-based methodologies are generally straightforward and cost-effective, allowing to prepare neat scaffolds or composites of different sizes. However, complex shapes can be hardly realized and a fine control of the final microstructure could not be ensured due to the random arrangement of porogens to be leached. The random configuration is also the typical result of the electrospinning process, which can be particularly suitable for the fabrication of soft tissue scaffolds, mimicking the specific ECM morphology. However, similar to the previous techniques, toxic solvents could be required as well, depending on the material to be processed, thus representing a safety risk to be accurately assessed, just like the high voltage needed for the electrospinning process. Such conditions can be regarded as a real limitation for a direct space application.

Going back to the scaffold random architecture, another consideration can be provided, with a special focus on 3D-printing. It is often reported that a regular pattern can be a valuable option, allowing to control the expected functionality and cell distribution^88^. This point could be of interest, but it should be critically revised as well, since tissue-engineered scaffolds have to be considered temporary substitutes for tissue-specific ECM, closely mimicking the microarchitecture. Generally, ECM is a complex structure not characterized by a regular and repetitive assembling of the key elements, e.g., collagen or elastin, and this morphology should be therefore resembled to promote a proper integration between the scaffold and the surrounding biological tissue to be healed. For this aim, thanks to the potential of 3D-printing, the design strategy of a tissue-engineered scaffold should be reviewed, including this requirement, in order to be as compliant as possible with a biomimetic approach^89,90^.

3D bioprinting, as already mentioned, seems to be the most promising technique to prepare a functional tissue-engineered construct in space. This approach has been tested so far mostly in parabolic flights, collecting cell-laden constructs with softer gels which are generally difficult to deposit on Earth due to the lack of self-standing capacity of such formulations^91^. The interest in the topic has currently promoted several attempts to develop bioprinters properly working in microgravity with the support of dedicated research programs from international space agencies, like ESA, NASA, and ROSCOSMOS^92^. However, the output of this manufacturing technique should be accurately designed with respect to the final application. Due to the intrinsic characteristics of the resulting scaffolds, with a particular focus on the mechanical properties, a number of possible limitations for the realization of biomimetic scaffolds for bone tissue engineering needs to be considered. Even by optimizing the material(s) to be bioprinted, the collected scaffolds are too soft as the compression modulus can be about 100 kPa, while that of bone is about 1 GPa^93,94^, and can unlikely provide the structural characteristics for load-bearing applications. In this respect, bioprinted scaffolds are mostly suitable to recover bone defects^95^.

## Bone tissue engineering in simulated microgravity: bioreactors

To understand the behavior of bone cells in long-term spaceflights, considering the limitations of direct cell exposure to real microgravity, ad hoc devices have been developed to simulate this particular condition. The term microgravity is commonly used irrespective of the real *g* value obtained from these devices, giving some differences between the various experiments carried out and also dealing with parameters not representative of different real microgravity conditions (e.g., Moon, Mars, extra orbital spaceflights). However, bioreactors provide useful information about the gravisensitivity of biological processes^96^. Table 3 summarizes the main microgravity simulation devices with their specific features.

**Table 3:** The main experimental devices to generate simulated microgravity.

Simulating device	Description	Output
2D-3D CLINOSTAT	The biological system is constantly rotated perpendicularly to the gravitational field. It is assumed that a biological system that rotates, with a repeatable pattern, around its center, being surrounded by liquid boundary, is not able to perceive gravity^130^. One rotation axis-2D clinostat Two rotation axis-3D clinostat	Vibration induced by the device can impact the collected results^131^.
ROTATING WALL VESSEL (RWV)	The biological system is kept in suspension, while it continuously falls within the fluid.	In order to minimize the centripetal acceleration, the sample should be as close as possible to the center of rotation. To deal with homogeneous results, a small number of samples in the reactor should be considered^132^.
RANDOM POSITIONING MACHINE (RPM)	The same technology as the clinostat, but here the speed and the direction are randomly changed during the experiment. The constant re-orientation of the gravity vector on an RPM does not allow biological systems to adjust to gravity^133^	The system produces small accelerations and vibrations that can be transmitted to cells, possibly damaging cellular structures^131^.

## Cells for bone tissue engineering

Various cell lines have been cultured as a model for studying bone cell biology, including primary cells from different species, induced osteoblasts from pluripotent stem cells and immortalized tumor cell lines. The use of these models presents advantages and disadvantages that should be considered before starting bone tissue engineering studies^97^.

The two principal cell lines used for bone tissue engineering are MSCs and adipose-derived stem cells (ADSCs), due to their genetic stability in long-term cultures and ability to differentiate in several lineages^98^. MSCs can be found in many different tissue sources within the body, including bone marrow and fat, while ADSCs can be harvested from adipose tissue. MSCs isolated from bone marrow have been found to have a high osteogenic potential, the ability to stimulate angiogenesis, and anti-inflammatory and immune-modulatory properties^99–101^; however, it is not trivial to harvest these cells from bone marrow^102^. ADSCs are the most used model thanks to their capability to differentiate into several cell types, being also characterized by the ease of accessibility and the stability in long-term cell cultures^103^.

Despite these two cell lines are characterized by good features for bone tissue engineering studies, human osteosarcoma cell line MG63, human primary osteogenic sarcoma cell line SAOS-2, and mouse osteoblastic cell line MC3T3-E1 are also generally used. MG63 and SAOS-2 present the advantage of the osteosarcoma cell lines to be available in unlimited number, showing an ease of maintenance, and a relative phenotypic stability as well. On the other hand, some reports highlight the evidence of progressing phenotypic heterogeneity, which is correlated with prolonged passaging of cells^104^. Additionally, this kind of cells do not reflect the whole range of phenotypic features of normal osteoblasts. MG63 cells respond to hormonal administrations like human osteoblastic cells, but have the disadvantage to arrest in the pre-osteoblast state. SAOS-2 show an expression profile to cytokine and growth factor-like human osteoblastic cells, but they do not mirror the range of phenotypic changes in human osteoblasts. MC3T3-E1 cells present phenotypic differentiation from preosteoblasts to mature osteoblasts, but also interspecies differences due to their nature^105^.

Along with these different properties, the selection of the appropriate and most relevant model for in vitro studies is critical, especially for some areas of medical research, such as pathological conditions (e.g., osteoporosis) in which the metabolic characteristics of the tissue depend not only from the disease but also from the senescence of the tissue.

## Bone tissue engineering onboard the International Space Station

The International Space Station (ISS) is a research laboratory orbits the Earth; both the vehicle and astronauts are in a constant state of free-fall. Considering the decade from 2011 to 2021, several experiments have been conducted on ISS with the aim to understand the molecular mechanism of osteopenia associated with the exposure of microgravity. Table 4 reports both the experiments carried out on the ISS and those previously performed on satellite launches^106^.

**Table 4:** Experiments performed in space related to osteopenia spaceflights.

Experiment name (acronym)	Brief description	Mission (date)
EDOS EDOS-1 EDOS-2	Early detection of osteoporosis in space. From 2009 in progress	ISS increment 19–20 (2009), 21–22 (2009), 29–30 (2011), 31–32 (2012), 33–34 (2012), 43–44 (2015), 45–46 (2015), 47–48 (2016), 49–50 (2016), 51–52 (2017),59–60 (2019), 61–62 (2019), 63 (2020), “Horizons” (2018).
ARED	Kinematics-biomechanical quantification of bone and muscle loading to improve the quality of µg countermeasure prescriptions for resistive exercise From 2011 in progress	ISS increment 47–48 (2016), 49–50 (2016), 51–52 (2017), 53–54 (2017), 57–58 (2018), 59–60 (2019)
IN VITRO BONE	Effect of µg at the bone cell and tissue level Date: 2018	ISS increment 55–56 (2018)
OBLAST	Osteosarcoma cell culture in weightlessness: morphology and biochemical response Date: 1995–1997	Foton 10 (1995), Foton 11 (1997)
OBADIS	Effects of µg on osteoclast and osteoblast Date: 2007	Foton M3 (2007)
STROMA	Response to µg of adult stem cells and osteoprogenitor from bone marrow Date: 2006	ISS 12S (2006)

The aim of the EDOS experiment is to detect bone micro-architectural changes and to provide a better evaluation of the kinetics of bone loss recovery post-flight. Measurements include three-dimensional peripheral quantitative tomography (XtremeCT), dual-energy X-ray absorptiometry (DEXA), and analysis of bone markers (blood samples). This experiment demonstrates that the lower extremities of bones are most affected by spaceflight effects and also contributes to develop new diagnostic protocols and high-resolution scanners. The relevance of the latter goal can be effectively regarded as an interesting case of downstream technology transfer.

ARED (advanced resistive exercise device) experiment aims to maintain and evaluate the muscle and bone strength of astronauts onboard using a device that allows the crew to engage in resistive exercises.

IN VITRO BONE experiment provides a complete in vitro model to mimic the process of bone matrix deposition and remodeling in real microgravity conditions, using a controlled chemical environment and ensuring real-time control and characterization during the evolution of cell cultures.

OBLAST experiment focused on the proliferation and morphological cell changes in weightlessness environment. For these experiments, osteosarcoma cells, isolated from rats, were used. The cells were inoculated with impaired cell cycle progression to evaluate if the morphological changes were related or not to the cell cycle stages.

OBADIS experiment aimed to study the effect of weightlessness on bone-forming cells (osteoblasts) and bone-degrading cells (osteoclasts).

In the STROMA experiment, bone marrow stromal cells (BMSCs), a cell population derived from bone marrow-containing progenitor cells for osteoblasts, were seeded onto porous biomaterials (bioceramics) to study the cell response to microgravity. The effects of microgravity on BMSCs were investigated by looking at morphological changes, gene expression profiling, and bone-forming capacity.

The above-reported experiments, except ARED, which evaluates possible countermeasures to the space environment, were carried out to investigate the complex cellular mechanism occurring in the remodeling process in microgravity. It should be emphasized that all these studies are fundamental not only to understand the physiological mechanisms in microgravity, but also to develop new technologies to be made available on Earth (downstream). Moreover, the variety of experiments shows the interest of space agencies (e.g., ESA and NASA) to better understand and define an effective approach to manage the complex scenario of the physiopathological changes affecting the musculoskeletal system in microgravity and contribute to safely plan long-term spaceflight missions.

## Overview of scaffold-based bone tissue engineering in simulated microgravity

In order to identify innovative solutions in response to the scientific needs in the field of bone tissue engineering in microgravity, and therefore overcome the limitations of hostile spatial conditions, it is necessary to carry out a detailed and targeted study on published data aimed to propose tailored experimental protocols. Over the years, works of increasing complexity have been presented, showing how bone cells react to an environment with reduced gravity.

Nishikawa et al. (2005) studied the osteogenic capacity of the rat marrow mesenchymal cells (MMCs) in vivo after a period of microgravitational culture. MMCs were cultured both in microgravity using a 3D clinostat, for an SMG value of 10^−3^ g, and in standard gravity conditions. After two weeks, cells were seeded onto an interconnected porous calcium hydroxyapatite scaffold and then implanted in vivo in rats for 8 weeks. All implants supported bone formation; however, the volume of newly formed bone was significantly lower for the clinostat group compared to the control group. These results indicate that new bone formation was inhibited in the SMG environment, due to the suppression of osteoblastic differentiation of MMCs^107^, highlighting a reduced osteogenic potential. This negative outcome showed an opposite trend with respect to other investigations, as reported below, anticipating, as a preliminary consideration, the advisability to move toward a homogenous and agreed approach to perform tailored experimental analyses. This point can be further supported by the intrinsic accessibility constraints to space, underlining, therefore, the need to optimize the research efforts. Indeed, a few years later, Jin et al. reported a study in which rat bone marrow mesenchymal stem cells (BMSCs) were cultured on ceramic bovine scaffolds in an SMG environment and then implanted into rat cranial bone defects. The experiment was carried out with two groups of samples: one under static conditions and the other under SMG conditions provided by a rotary wall vessel (RWV). After two weeks of culture, osteogenesis in both groups was verified by performing DNA and alkaline phosphatase (ALP) analysis. The authors reported that DNA content and ALP were higher for cells grown on SMG conditions, indicating better viability. They also reported the presence of bone matrix inside the scaffolds. The two groups of engineered bone constructs were then implanted into Sprague-Dawley rat cranial bone defects. After 24 weeks, the engineered bone constructs under dynamic culture were found to repair the defects better, as shown by an enhanced histological bone connection. This may be due to the dynamic conditioning provided by the RWV, which represents a useful in vitro model for reproducing the functional role of osteogenesis in BMSC proliferation, differentiation and maturation^108^.

Araujo et al. published a study on human osteosarcoma-derived cells cultured in RWV reactors for two weeks using two different types of electrospun scaffolds. The first type was made up of biomimetic calcium phosphate–coated poly(caprolactone) nanofiber meshes (BCP-NMs) and the other one was produced with only poly (caprolactone) nanofiber meshes (PCL-NMs). Scaffolds were defined as biomimetic because the calcium phosphate coating mimicked the bone composition, thus improving the scaffold-bonding capability of the cells. It was verified that BCP-NMs samples cultured in RWV presented a more massive production of proteins associated with the bone extracellular matrix^109^. This result focused for the first time on the realization of biomimetic scaffolds, even if biomimicry only concerned the material composition and not the morphology or the mechanical properties. The value of simulated microgravity in this experiment was not specified.

**Fig. 3:**
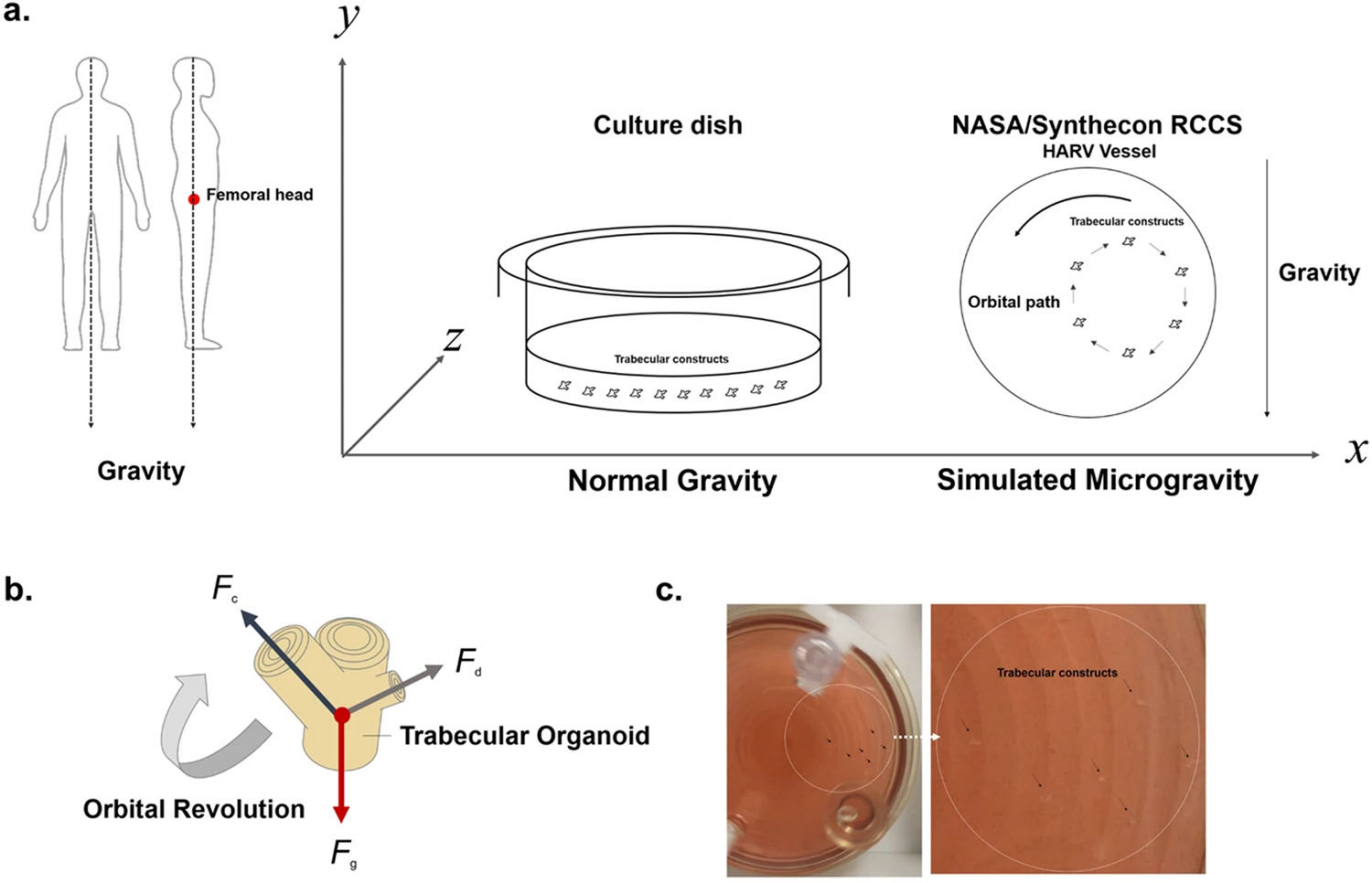
Scaffold-based simulated microgravity assay. Sketch of the experimental conditions showing both static and dynamic models, the latter being characterized by a laminar movement of the culture medium suspending constructs in a state of orbital buoyancy (**a**), forces acting on the organoid during each orbital revolution (**b**), preventing sedimentation and simulating weightlessness (**c**) (modified from ref. ^110^, in compliance with CC BY 4.0 License—https://creativecommons.org/licenses/by/4.0/).

The interest in cell cultures seeded on scaffolds in simulated microgravity requires more complex studies to effectively evaluate proliferation, adhesion, migration, and the ability to form the cytoskeleton. In this regard, it is pivotal to deal with a 3D scaffold that closely mimics the physiological environment. Avitabile et al. assessed the behavior of human bone marrow-derived mesenchymal stem cells seeded on a magnesium-doped nanocrystalline hydroxyapatite/type I collagen composite scaffold (MgHA/Coll) in a random positioning machine, reporting a generic 0-*g* value. Collected data showed that, after 3 weeks in SMG, nanostructured MgHA/Coll scaffolds can alleviate μg-induced osteoblast dysfunction, promoting cell differentiation along the osteogenic lineage, with a consequent reduction in the expression of some surface markers^40^. These results highlighted how composite scaffolds promoted osteogenesis even in extreme μg long-term conditions, suggesting the possibility to deal with advanced substrates to counteract bone loss. Dealing with biologically-derived scaffolds, Iordachescu et al. prepared a micron-scale bone organoid prototype using human osteoblasts cells to be seeded onto trabecular bone fragments from bovine femurs. The construct was firstly encapsulated in human fibrin and then located into a NASA simulated gravity bioreactor (value of microgravity not specified) for 5 days. Under SMG conditions, the organoids provided a model of the pathological state of reduced mechanical stimulation and osteoclastic bone resorption sites with a morphology different from that of the static control were detected; a scheme of the experimental assay is shown in Fig. 3.

It was also observed that bone fragments were lost from the initial culturing structures, further simulating the bone loss process^110^. From this work, a deep understanding of bone degradation mechanism in reduced gravity can be addressed by specifically engineering scaffolds toward biomimetic constructs both from a compositional and morphological point of view. Table 5 briefly summarizes the analyzed reports.

**Table 5:** Summary of the collected biological responses in simulated microgravity.

Report	μg level (g)	μg System used	Scaffold	Cell line	Time in μg (weeks)	Results
The effect of simulated microgravity by three-dimensional clinostat on bone tissue engineering^107^	10^–3^	3D clinostat	Interconnected porous calcium hydroxyapatite	Human marrow mesenchymal cells	2	Microgravity hinders cell growth
Establishment of three-dimensional tissue-engineered bone constructs under microgravity-simulated conditions^108^	Not specified	RWV	Ceramic bovine bone	Rat bone marrow mesenchymal stem cells	2	Hydrodynamic microgravity conditions in tissue-culture bioreactors can modulate the composition, morphology, and function of the engineered bone
Dynamic culture of osteogenic cells in biomimetically coated poly(Caprolactone) nanofiber mesh constructs^109^	Not specified	RWV	BCP-NMs	Human osteosarcoma-derived cell line	2	Scaffolds appeared to be a supportive means for osteogenic cell growth and ECM production
Bioinspired scaffold action under the extreme physiological conditions of simulated spaceflights: osteogenesis enhancing under microgravity^40^	Not specified	Random position	MgHA/Coll	Human bone marrow-derived mesenchymal stem cells	1, 2, 3	Improved differentiation into osteogenic cells under static conditions and restored the osteogenic differentiation after dysregulation induced by μg
Trabecular bone organoids: a micron-scale “humanized” prototype designed to study the effects of microgravity and degeneration^110^	Not specified	RWV	Trabecular bone fragments from bovine femurs	Human osteoblasts from a female gender	0,7	Production of bone ECM supported by biologically-derived scaffolds

## Future outlook and summary

Bone tissue engineering in microgravity is a key-research topic for human space exploration. In this regard, cell-scaffold interaction is fundamental for a successful healing process and a suitable strategy can be developed by mimicking bone ECM as much as possible. A potential approach can be the design and fabrication of bioactive nanocomposite scaffolds to improve the mechanical and biological properties, as this solution might support a promising outcome. It should be underlined, however, that special attention should be paid to the cell culture protocols as the variability of the SMG systems may lead to different results depending on the equipment used. It seems, therefore, reasonable to suggest a standardization of the experimental protocols toward an agreed consensus in order to deal with easy comparable data. Moreover, different *g* values should be investigated when planning missions to Moon (0.16 g) and Mars (0.37 g). Obviously, testing tissue-engineered constructs in real microgravity would be the desirable condition, but, given the limited access to space, a comprehensive study design should be developed to define the next experimental approach, providing more reliable results and defining shareable requirements for investigations in space laboratories.

From a perspective point of view, research in space offers the opportunity to enhance the knowledge on bone tissue impairment in a very harsh environment with the possibility to extend the results to clinical practice on Earth, as a subsequent outcome to modify or introduce different and/or alternative investigation models. For instance, referring to the osteoporotic condition, the research in microgravity might offer an experimental opportunity to validate, and then further enhance, the downstream/upstream relationship to highlight and compare possible similarities, develop novel pathophysiological in vitro models, and assess alternative and improved therapeutical protocols. Concerning bone tissue engineering in space, with a special focus on scaffold-based studies, a potential approach to be developed should include the biomimetic design of three-dimensional cell culture substrates. This topic can ensure a more reliable biological response supported by tissue-specific models that can actually provide a significant cell-scaffold (i.e., ECM replicas) interaction. Indeed, several research areas can benefit from this scenario, as reported by the International Space Station National Laboratory, for, e.g., modeling human diseases based on accelerated case studies of aging or pathologies; growing 3D cell cultures, as in microgravity complex three-dimensional structures are formed providing suitable models to study cell behavior, advance regenerative medicine, and test the effects of new drugs; or elucidating molecular mechanism to reveal new information about how specific groups of genes influence the biological response and understand fundamental mechanisms involved in human health and disease.

### Reporting summary

Further information on research design is available in the Nature Research Reporting Summary linked to this article.

## Supplementary information

Reporting Summary
